# Easy and green synthesis of nano-ZnO and nano-TiO_2_ for efficient photocatalytic degradation of organic pollutants

**DOI:** 10.1016/j.heliyon.2024.e37469

**Published:** 2024-09-05

**Authors:** Nusrat Jahan Tamanna, Md. Sahadat Hossain, Sumaya Tabassum, Newaz Mohammed Bahadur, Samina Ahmed

**Affiliations:** a Glass Research Division, Institute of Glass & Ceramic Research and Testing, Bangladesh Council of Scientific and Industrial Research (BCSIR), Dhaka, 1205, Bangladesh; b Department of Applied Chemistry and Chemical Engineering, Noakhali Science and Technology University, Noakhali, Bangladesh

**Keywords:** Radical scavenger, Industrial effluent, Nanocomposite, Photocatalysis, Congo red

## Abstract

As the textile industry expands, more industrial waste effluents are released into natural water streams, prompting the research and development of innovative materials for the remediation of environmental issues. In this research, a direct precipitation and hydrolysis method were used to synthesize ZnO and TiO_2_ nanoparticles, respectively that were utilized to investigate the photocatalytic activity of Congo Red (CR) dye. Afterward, the crystallite size was computed from the data of the X-ray diffractometer (XRD), and utilizing several models (Scherrer equation, LSLMSE, Monshi-Scherrer equation, Williamson-Hall model, Size-strain plot method, Halder-Wagner model, Sahadat-Scherrer model). Among these models, the size-strain plot model yields the most accurate crystal size (45.31 nm) for ZnO nanoparticles and the Halder-Wagner model (2.44 nm) for TiO_2_ nanoparticles. Scanning Electron Microscope exhibited the spherical shape of nanoparticles (ZnO, and TiO_2_) with particle size (less than 151 nm). The absorption spectrum from Fourier transform infrared (FTIR) spectroscopy confirmed the formation of nanoparticles (ZnO, and TiO_2_). Thereafter, the photocatalytic activity of the ZnO-TiO_2_ nanocomposite was evaluated by using Congo Red (CR) dye under different process variables, such as catalyst dose, time, initial dye concentration, pH, radical scavenging ability, and reusability. The best degradation (90 %) was recorded at 180 min time intervals using a 0.2 g catalyst dose with a 20 ppm CR concentration at pH 9.

## 1 Introduction

With an increasing population, the industry demand has also risen worldwide, creating immense pressure on the planet Earth's environment and causing various types of pollution, such as water pollution, air pollution, land pollution, and noise pollution [1]. Among these, water pollution is considered a major source of pollution, as industrial, municipal, and sewage waste is discharged into natural water streams. The major pollutants found in industrial wastewater include organic pollutants (organic acid, phenol, detergent, etc.) [2,3]; inorganic pollutants (sulphates, phosphates, salt, bleaches, etc.) [4]; suspended solids [5]; and microorganisms [6]. Additionally, more industrial waste effluents containing pollutants, particularly organic pollutants from the pharmaceutical, pulp, paper, RMG, and textile sectors, are being dumped into adjacent water systems as a result of industrialization [7]. A report from the United Nations stated that more than 80 % of wastewater worldwide is disposed of into the environment without any sort of purification, while in many developing nations, this percentage even surpasses 95 % [8]. The World Bank estimates that the wastewater produced by the department responsible for dyeing and finishing comprises between 17 and 20 % of all industrial effluent. Textile dyeing is the second largest water polluter in the world and accounts for 20 % of the wastewater produced worldwide [9]. Chemical contaminants prevalent in textile wastewater include starch, acid, chromium, and dyes. Approximately 3,00,000 tons of synthetic dyes are reportedly discharged into treatment facilities worldwide each year [10].

In the present study, ZnO nanoparticles with distinct chemical and physical characteristics were synthesized [11,12]. Among the various other methods for synthesizing ZnO, the direct precipitation method was chosen for further study. Zn(NO_3_)_2_·6H_2_O, the precursor ingredient for the procedure, and KOH react with one another while being vigorously stirred to produce a white precipitate. For synthesizing nano-TiO_2,_ the precursor material was vigorously stirred after mixing with solvents. After perfect mixing, the TiO_2_ that formed a gel was filtered, subsequently dried, and finally calcined to form nanosized anatase TiO_2_.

A wide variety of materials with at least one dimension between 1 and 100 nm make up the unique class of materials known as nanomaterials [13,14]. In contrast to their bulk analogs, nanomaterials exhibit certain distinctive features, including mechanical, electrical, thermal, optical, magnetic, and catalytic features [15]. It is thought that the small size of nanomaterials is the main cause of their distinct characteristics. It is possible to create exceptionally large surface areas with nanomaterials [16]. Although research on group II-VI semiconductor nanomaterials has been ongoing for several decades, most related research has focused on low-dimensional metal oxide structures [[17], [18], [19]]. A metal oxide of group IIB, ZnO, has been extensively studied and documented due to its superior nanoscale applications, including in photovoltaic devices (gas sensors, solar cells, field effect transistors), optoelectronics, and biological labeling [[20], [21], [22]]. Zinc oxide is one of the most versatile metal oxide semiconductors. Another semiconductor nanomaterial that has been investigated is TiO_2_. This unique feature of these nanomaterials is because of their chemical stability, low toxicity, photocatalytic activity, and ease of manufacturing [23,24]. Titania has been applied extensively for treating organic pollutants in wastewater, where it functions as a photocatalyst to degrade these pollutants [25]. Titania exists in two main phases: rutile (a tetragonal phase) and anatase (a tetragonal phase). Among these two phases, the anatase phase is the most catalytically active [26,27].

The majority of dyes used in industry are hazardous and carcinogenic, endangering both human health and the marine environment [28]. These dyes severely degrade the quality of water bodies, as they increase the levels of BOD and COD, hinder photosynthesis, impede plant growth, infiltrate the food chain, cause bioaccumulation and recalcitrance, and may be toxic and mutagenic [29]. Therefore, it is crucial to implement treatment techniques using physical, chemical, or biological technologies or a combination of these methods to assure the sustainability of the environment for future generations. Despite being effective, these methods have drawbacks, such as the difficulties associated with disposing of sludge, the high cost of power, and high operation costs. As a result, a more effective technique called photocatalytic degradation has been developed. The use of photocatalytic technology has demonstrated significant promise as an affordable, environmentally friendly, sustainable solution for completely removing pollutants without causing any secondary pollution [30,31]. Nanoscale catalysts have enormous potential for photocatalytic activity and are therefore capable of degrading different types of dyes discharged from the industry.

In this research work, the photocatalytic activity of synthesized ZnO-TiO_2_ nanocomposites was elucidated to degrade the model dye Congo Red. Yet ZnO and TiO_2_ nanoparticles have high photocatalytic activity on their own; this work attempted to demonstrate how their activity boosted when blended as a nanocomposite. The main goal was to achieve excellent performance of the photocatalyst, which can be observed through the optimization of different variables. Furthermore, XRD, FTIR, and SEM analyses were conducted to confirm the presence of the nano photocatalyst.

## 2 Materials and methods

### 2.1 Materials

The major chemical used for the synthesis of zinc oxide nanoparticles was zinc nitrate hexahydrate [Zn(NO_3_)_2_.6H_2_O, 98 % (purchased from “Fluka”)]; KOH from Merck, Germany; and DI water. Additionally, the chemicals used for synthesizing titanium dioxide nanoparticles were tetrabutyl orthotitanate [C_16_H_36_O_4_Ti (purchased from Tokyo Chemical Industry Co. Ltd.], ethanol (C_2_H_5_OH), nitric acid (HNO_3_) and DI water.

### 2.2 Methods of preparation

#### 2.2.1 Synthesis of zinc oxide nanoparticles

This study employed a direct precipitation approach to produce zinc oxide nanoparticles from the reaction of [Zn(NO_3_)_2_.6H_2_O] with KOH. The initial step was to prepare a 0.2 M aqueous solution of [Zn(NO_3_)_2_.6H_2_O]. For this, 10 g Zn(NO_3_)_2_.6H_2_O was taken into a 1000 mL beaker, into which 170 mL DI was added. Next, 0.4 M aqueous KOH solution was made by adding 170 mL DI into 3.77 g KOH. The pH was maintained above 12. The KOH solution, which functions as a precipitating agent, was then gently introduced from the burette into the zinc nitrate solution while constantly stirring. The formation of a white suspension proved that the reaction was complete. The reaction mixture was then filtered using Whatman filter paper after three washes with DI water and one with ethanol. In the end, the precipitate product was calcined at 500 °C for 3 h following a 2 h oven drying time at 105 °C, and the overall chemical reaction is expressed by equation (1) [32].

(1) Zn(NO_3_)_2_.6H_2_O + 2KOH = ZnO + 7H_2_O + 2KNO_3_

#### 2.2.2 Synthesis of titanium dioxide nanoparticles

The hydrolysis method was used for synthesizing titanium dioxide from tetrabutyl orthotitanate. The first step of the process involved hydrolyzing C_16_H_40_O_4_Ti in a mixture of HNO_3_, C_2_H_5_OH, and DI water. A ratio of 1:15:4:3 was maintained for C_16_H_40_O_4_Ti, C_2_H_5_OH, DI water, and HNO_3_, and mixing was carried out to form a sol of TiO_2_ under vigorous stirring for 5 h. The reaction medium was maintained as acidic. Sol that formed a gel when standing overnight was then dried at 80 °C for 2 h after filtration. Finally, calcination at 200 °C for 2 h in the furnace provided the desired anatase form of TiO_2_ [33].

### 2.3 Characterizations

#### 2.3.1 UV–visible spectroscopic analysis

To measure the absorbance of the ZnO and TiO_2_ nanocomposites, a UV–visible spectrometer (model U-2910) with a wavelength range of 450–550 nm was used. A unique type of quartz cell with a width of 1 cm was utilized as the cuvette. UV–visible irradiation was provided by deuterium and tungsten lamps. The samples were collected in aqueous media, and the device was calibrated using DI water.

#### 2.3.2 X-Ray diffraction analysis

The instrument used to perform the crystallographic phase analysis of the synthesized nanoparticles was a Rigaku SE instrument with a scan range of 5°–70° and a step width of 0.01. As a radiation source, a ceramic copper tube (Cu Kα) was chosen for this study because of its wavelength of 1.5406 Å and accelerating voltage and current of 40 kV and 50 mA, respectively. The operating temperature of the cooling water system was maintained at 19–20 °C. By calibrating the instrument using the standard silicon reference, the accuracy of the instrument was evaluated. The ZnO and TiO_2_ nanoparticle data corroborate the data obtained from the International Centre for Diffraction Data (ICDD) database, with card numbers #00-036-1451 and # 01-075-2544.

#### 2.3.3 FTIR spectrometric analysis

The distinctive functional groups present in the FTIR spectra of the ZnO and TiO_2_ nanoparticles were investigated by using an FTIR spectrophotometer (IR-Prestige 21, Shimadzu, Japan) with attenuated total reflection (ATR) attachment. The transmittance mode was kept between 400 and 4000 cm^−1^ with a spectral resolution of 4 cm^−1^ to obtain the data. The data are presented as the average of 30 scans.

#### 2.3.4 Photocatalytic activity

Materials that undergo photocatalytic reactions when exposed to photoirradiation are known as photocatalysts. Semiconductor materials, viz., ZnO and TiO_2,_ are considered the most prominent candidates for use as photocatalysts due to the presence of a valence band and conduction band in their electronic structure. As a result of having a valence and conduction band, they are capable of exhibiting photocatalytic activity when light (UV–visible light, IR irradiation, etc.) of different wavelengths falls onto them, which creates electron-hole pairs due to excitation. Therefore, photocatalytic activity may be termed the ability of a photocatalyst to generate electron-hole pairs that initiate free radicals and further secondary reactions. This research describes a detailed analysis of the photocatalytic activity of ZnO-TiO_2_ nanocomposites utilizing a halogen lamp under specific operating conditions for a 0.2 g sample with a ZnO: TiO_2_ ratio of 1:1, 40 mL of 20 ppm dye (Congo red dye), a 180 min time and room temperature. Equation (2) depicts the photodegradation percentage equation of the nanocomposite based on the absorbance measured with a UV–visible spectrophotometer, where C_0_ represents the initial dye concentration and C_t_ represents the final dye concentration at time t.

(2) $$\text{Photodegradation}\;\text{percentage},P_{p}=\frac{Co-Ct}{Co}\times 100$$

Equation (3) represents the degradation capacity, where the amount of dye degradation was calculated by the absorbance obtained from a UV–visible spectrophotometer. Here, C_0_ and C_t_ represent the initial and final dye concentrations, respectively; W represents the weight of the sample in grams (g); and V represents the volume of dye solution in the literature.

(3) $$\text{Photodegradation}\;\text{capacity},q_{e}=\frac{Co-Ct}{W}\times V$$

## 3 Results and discussion

### 3.1 Crystallographic analysis

**Fig. 1:**
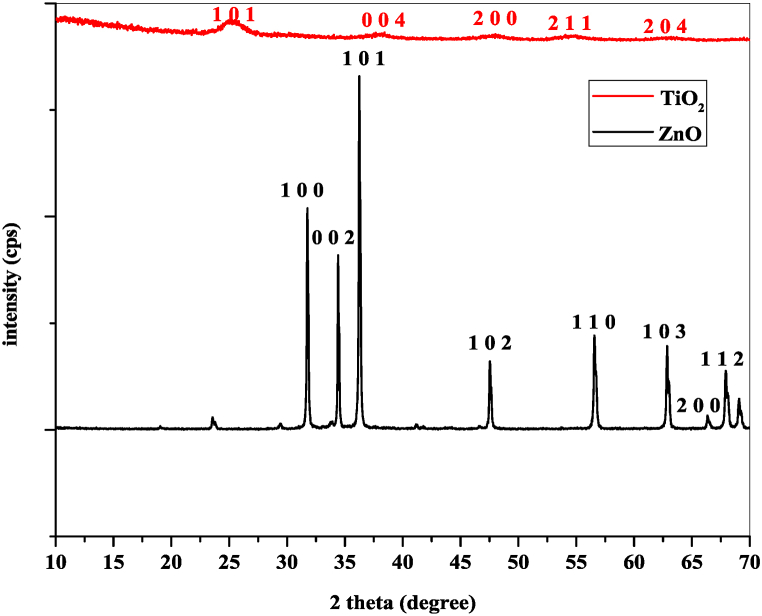
XRD peak pattern for synthesized ZnO and TiO_2_.

Fig. 1 shows the XRD patterns of the synthesized ZnO and TiO_2_ nanoparticles, where the peak intensities in cps and 2 θ are on the y-axis and x-axis, respectively. From the diffraction pattern, the characteristic peaks for the ZnO nanoparticles appeared at 31.7637°, 34.4212°^,^ and 36.2414° 2 θ positions, with equivalent planes at the (100), (002), and (101) planes, respectively. All these data complement the data from the standard ICDD database, which includes card no. 00-036-1451 and has a hexagonal crystal structure that confirms the formation of nanoparticles. However, the diffractogram of the TiO_2_ nanoparticles revealed the largest peaks at 25.34°, 38.10°, and 47.82° 2 θ positions, with corresponding planes at (101), (004), and (200), respectively, which matched the standard ICDD data of card no. #01-075-2544.

A variety of XRD models for determining the crystal size and microstructural parameters, such as the lattice strain, stress, and lattice energy density, are available. These included the Scherrer equation, the linear straight-line method of the Scherrer equation (LSLMSE), the Sahadat-Scherrer model, the Monshi-Scherrer equation, and the Williamson-Hall method [including the uniform deformation model (UDM), uniform stress deformation model (USDM), uniform deformation energy density model (UDEDM), the size-strain plot method (SSP), and the Halder–Wagner method]. All of these model equations for estimating crystal size have been thoroughly explained elsewhere [[34], [35], [36], [37]]. Table 1 shows the crystallite size of the synthesized nanoparticles according to the aforementioned models.

**Table 1:** Crystal sizes of ZnO and TiO_2_ nanoparticles according to various models.

Model	ZnO, D (nm)	TiO_2_ (anatase), D (nm)
Scherrer	792.89	38.17
LSLMSE	1540.6	22.36
M-S	61.29	0.64
UDM	69.67	3.03
USDM	58.01	3.03
UDEDM	57.72	3.03
SSP	45.31	3.72
H-W	72.46	2.44
S‒S	770.3	4.33

### 3.2 FTIR analysis

For both organic and inorganic materials, FTIR provides quantitative and qualitative information. An infrared absorption spectrum is produced using Fourier transform infrared (FTIR) spectroscopy, which is used to determine the chemical bonds of a molecule. The profile of a sample, which is created by spectral information, is a unique molecular fingerprint that may be used to screen and scan samples for a variety of constituents. To identify functional groups and describe covalent bonding information, FTIR is a useful analytical tool.

**Fig. 2:**
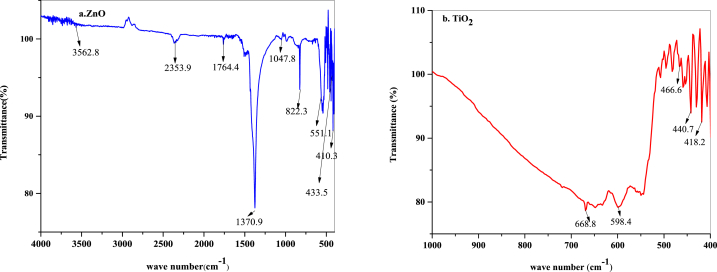
FTIR absorption peaks for a. ZnO and b. TiO_2_.

Fig. 2 (a) and 2 (b) show the FTIR spectra of the synthesized ZnO and TiO_2_ nanoparticles for identification of the functional groups present in the ranges of 400–4000 cm^−1^ and 400-1000 cm^−1^, respectively. In the case of zinc oxide nanoparticles, the absorption peaks were expected to occur at 410.3 cm^−1^, 551.1 cm^−1^, 822.3 cm^−1^, 1047.8 cm^−1^, 1370.9 cm^−1^, 1764.4 cm^−1^, 2353.9 cm^−1^ and 3562.8 cm^−1^. The fingerprint region of the zinc oxide nanoparticles appeared in the range of 1500–600 cm^−1^ [38]. The Zn-O absorption peak (stretching vibration) occurs between 410.3 and 551.1 cm^−1^. The C-N bond of the amine or the C-O bond of the primary alcohol was generated by stretching vibrations at 1047.8 cm^−1^. The O-H stretching vibration gives rise to an absorption peak at 3562.8 cm^−1^. Moreover, for the anatase titanium dioxide nanoparticles, the major absorption peaks appeared at 418.2 cm^−1^, 440.7 cm^−1^, 466.6 cm^−1^, 598.4 cm^−1^, and 668.8 cm^−1^. The absorption peaks in the range of 400–700 cm^−1^ confirm the stretching and bending vibrations of Ti-O-Ti [39].

### 3.3 Photocatalytic activity analysis

#### 3.3.1 Photocatalyst dose variation

**Fig. 3:**
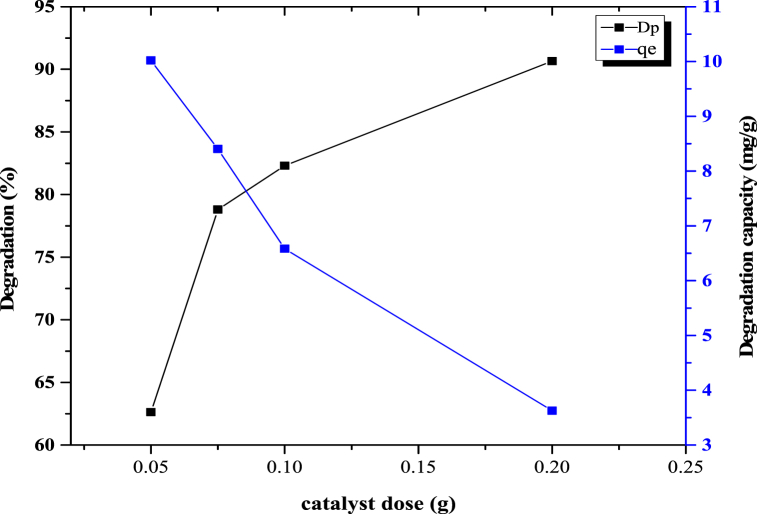
Schematic representation of different photocatalyst doses.

While performing the photocatalytic activity experiments, different photocatalyst doses were found to result in different degradation percentages and capacities. It was very important to identify which photocatalyst dose provided the best results. As a consequence, various dosages of synthesized photocatalysts were chosen to study the degradation percentage and capacity of the dyes. This experiment was carried out for a ZnO-TiO_2_ nanocomposite photocatalyst using dosages of 0.5 g, 0.75 g, 0.1 g, and 0.2 g over a 180 min time with 20 ppm, 40 mL of Congo red dye. Fig. 3 shows the degradation percentage and capacity against the photocatalyst dose determined by using Origin software. It is evident from the figure that the degradation percentage increased with decreasing photocatalyst dosage, while the degradation capacity decreased with increasing dosage. A maximum of 90.65 % degradation occurred for the 0.2 g photocatalyst dose, whereas a minimum degradation of 62 % was recorded for the 0.1 g sample. In contrast, a maximum degradation capacity of 10.01 mg/g was recorded for the 0.1 g sample, but 0.2 g showed a minimum of 3.62 mg/g. A significant catalytic reaction occurred because the catalysis involved the adsorption and breakdown of Congo red dye, and the presence of additional catalysts in the solution increased the number of active sites.

#### 3.3.2 Effect of time variation

**Fig. 4:**
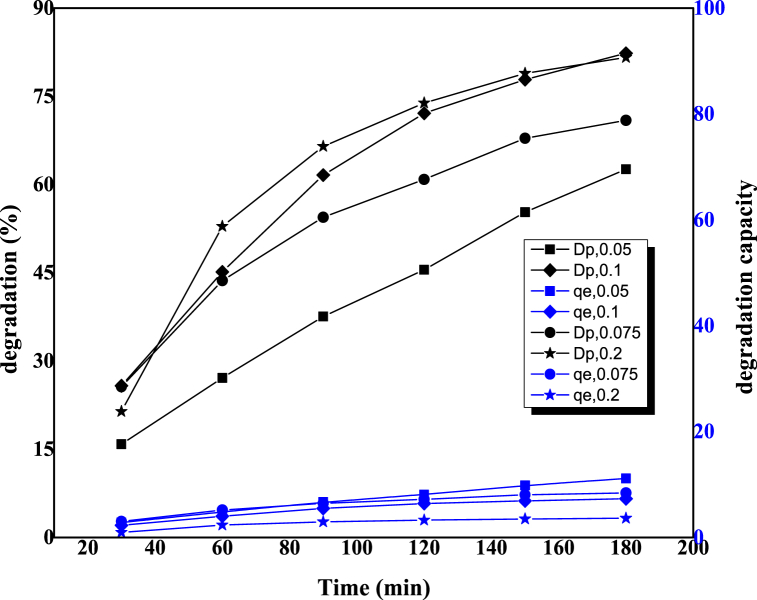
Schematic representation of the effect of different time.

This experiment was conducted because the photocatalytic activity of each sample varies with time. It was determined through the investigation of photocatalytic dosage variation that a 0.2 g sample yields the best degradation; hence, further experiments were carried out using this amount of sample. In Fig. 4, the degradation percentage and degradation capacity of the 0.2 g ZnO-TiO_2_ nanocomposite were evaluated over 30 min, 60 min, 90 min, 120 min, 150 min, and 180 min periods. Fig. 4 shows that both the degradation percentage and capacity of the sample increased with increasing time. A maximum of 90.65 % degradation occurred at 180 min with a 3.63 mg/g degradation capacity.

After the completion of this experiment, with time intervals of 180 min, the time was optimized for further studies with an optimized 0.2 g sample dose.

#### 3.3.3 Effect of dye concentration variation

**Fig. 5:**
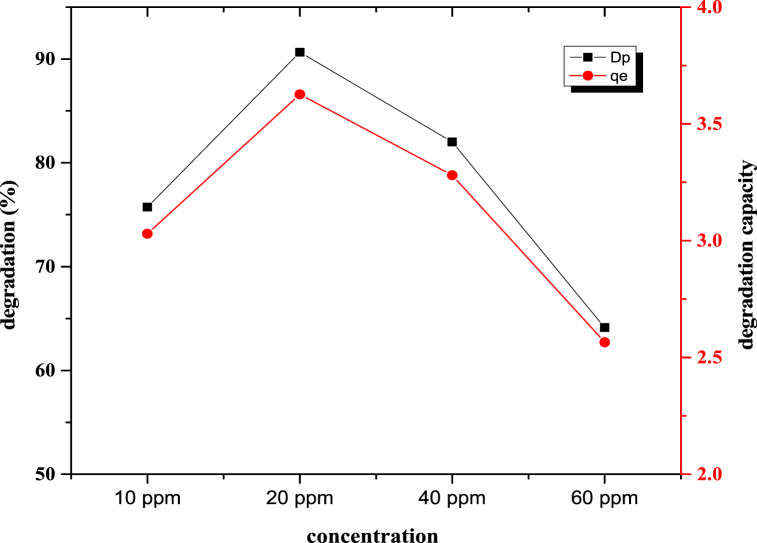
Schematic representation of the different dye concentrations.

Fig. 5 shows the variation in the degradation percentage and degradation capacity with different dye concentrations. This study involved the exposure of a 0.2 g ZnO-TiO_2_ nanocomposite to light for 180 min while varying the dye concentration to 10 ppm, 20 ppm, 40 ppm, or 60 ppm. The following figure shows that the initial degradation percentage and degradation capacity increased with increasing concentration. At 20 ppm, the highest percentage of degradation occurred, after which the degradation rate decreased with increasing concentration. A similar phenomenon occurred for the degradation capacity. A maximum 90.65 % degradation percentage was recorded at 20 ppm, corresponding to a 3.63 mg/g degradation capacity. This experiment showed that with increasing initial dye concentration, the dye's active site became more accessible, and as a result, the degradation percentage decreased.

#### 3.3.4 Effect of different pH ranges

**Fig. 6:**
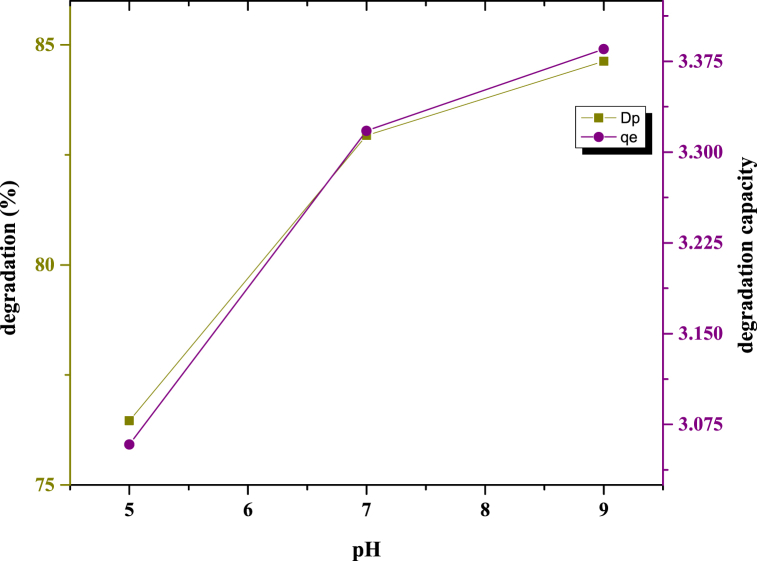
Schematic representation of the different pH ranges.

The photocatalytic activity of the synthesized nanocomposite was evaluated at different pH values, and Fig. 6 illustrates this phenomenon. The degradation percentage and degradation capacity were measured using a 0.2 g sample for 180 min at pH 5, pH 7, and pH 9. Acidic and basic pH were maintained by using an acidic nitric acid solution and a basic ammonium hydroxide solution, respectively. A lower pH was associated with the least amount of degradation. With increasing pH, both the degradation percentage and degradation capacity increased. At pH 9, the highest degradation percentage (84.6 %) and the highest degradation capacity (3.38 mg/g) were observed. This is because higher pH values of OH^−^ can result in the production of more hydroxyl ion radicals when exposed to light.

#### 3.3.5 Scavenger test

In chemical treatment, a scavenger is a chemical that is introduced into a mixture to eliminate or deactivate undesirable reaction products, such as oxygen and contaminants, to ensure that no adverse reactions occur [40]. While photocatalysis has emerged as a promising “green” technology for degrading organic pollutants, traditional physical adsorption and biodegradation treatments are easily deactivated and can result in secondary pollution [[41], [42], [43]]. Photocatalysis is an essential component of photocatalysis that involves photoinduction of electron-hole (e^−^-h^+^) pairs to start the breakdown of contaminants [44]. Therefore, the key to a highly effective photocatalytic process is the use of a well-designed photocatalyst.

Free radicals, or simple ROS, are substances or minute particles with single electrons in their atomic or molecular orbitals. They also include a wide variety of oxygen species, such as hydrogen peroxide and hydroxyl groups. Reactive oxygen species (ROS) are produced during UV-induced photocatalysis, although hydroxyl radical production is often linked to the breakdown of persistent organic pollutants in water. A range of radical scavengers have been used to distinguish the functions of various ROS. In this study, to examine the reactive species responsible for CR degradation, several scavenger tests were conducted, including those involving IPA, EDTA, and DPPH for hydroxyl, hole, and free radicals, respectively. The charge carriers can move toward the catalyst surface and start redox reactions. In these reactions, hydroxyl radicals (•OH) can be produced by the oxidation of OH or water adsorbed at the surface by h^+^ VB, while superoxide radical anions (O_2_^•−^) can be generated by scavenging e^−^ CB by adsorbed molecular oxygen. O_2_^•−^ can further react to generate singlet oxygen and hydrogen peroxide, which can then yield •OH [45]. At surface trapping sites or in the bulk of a catalyst, interfacial charge transfer from photogenerated electrons or holes to acceptor/donor species at the surface competes with charge recombination. Nonradiative decay processes cause the electron/hole pair to recombine, releasing heat and releasing the adsorbed photonic energy. Because photocatalysis produces reactive oxygen species (ROS) when exposed to light, it is a vital tool for killing pathogenic microorganisms and remediating persistent organic pollutants in water and air. Its environmentally benign qualities also include its nontoxicity, photostability, and lack of dissolution in water.

A well-known, rapid, simple, and cost-effective method for measuring catalytic degradation is 2,2-diphenyl-1-picrylhydrazyl (DPPH), which uses free radicals to determine whether a material can act as a hydrogen supplier or a free-radical scavenger (FRS). The elimination of DPPH, which is a stabilized free radical, is related to the DPPH testing procedure. An odd electron combines with the free radical DPPH to produce a significant absorbance at 517 nm or a purple color [46].

#### 3.3.6 Radical scavenger test using isopropyl alcohol

**Fig. 7:**
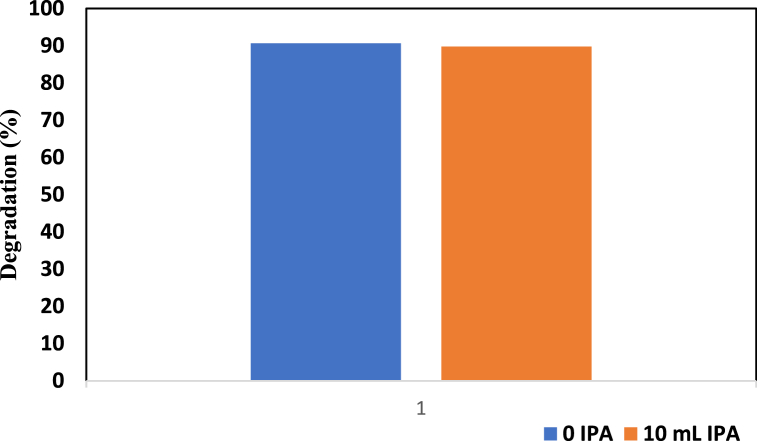
Radical scavenger test using 10 mL IPA for the ZnO-TiO_2_ nanocomposite.

IPA was tested for radical scavenging ability under standard conditions for the ZnO-TiO_2_ nanocomposite photocatalyst. To perform this experiment, 0.2 g of catalyst was exposed to light for 180 min, and the dye concentrations used were 20 ppm and 40 mL. Afterward, 10 ppm (10 mL) IPA solution was added to the dye solution. Fig. 7 demonstrates this phenomenon. As shown in Fig. 7, the addition of IPA decreased the degradation percentage of the sample, as a degradation percentage of 89.78 % was associated with 10 mL IPA.

#### 3.3.7 Hole scavenger test using EDTA

**Fig. 8:**
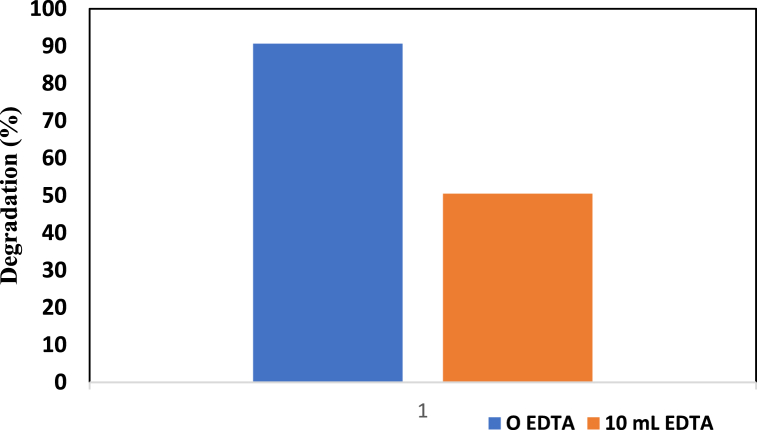
Hole scavenging test of ZnO-TiO_2_ nanocomposites using 10 mL of EDTA.

To investigate the importance of the hole scavenger agent, ethylenediaminetetraacetic acid (EDTA) was utilized under standard conditions. This test was accomplished by using a 0.2 g sample with a 20 ppm (40 mL) dye concentration for 180 min. Later, by adding 10 ppm (10 mL) EDTA solution to the abovementioned solution, the degradation percentage was determined. Fig. 8 indicates that the percentage of degradation decreased when EDTA was added. A degradation of 50.52 % was noted, which is much lower than the standard degradation.

#### 3.3.8 Free radical scavenger test using DPPH

**Fig. 9:**
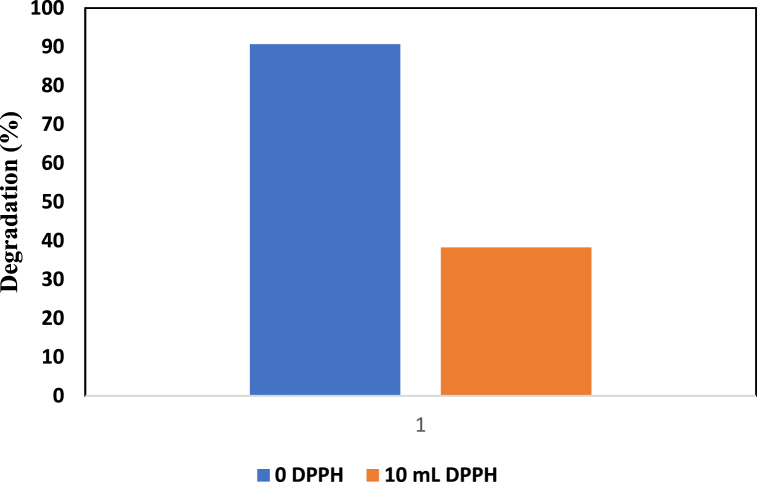
Free radical scavenging test using 2 mL of DPPH solution for the ZnO-TiO_2_ nanocomposite.

Using 2,2-diphenyl-1-picrylhydrazyl (DPPH) under normal conditions, a free radical scavenging test was carried out. While performing the test, 2 mL of DPPH solution was added to a 0.2 g ZnO-TiO_2_ photocatalyst and exposed to light for 30 min. Fig. 9 shows that the degradation percentage decreased with the addition of DPPH. When DPPH was added, the deterioration rate decreased to 38.25 %.

#### 3.3.9 Reuse ability test

**Fig. 10:**
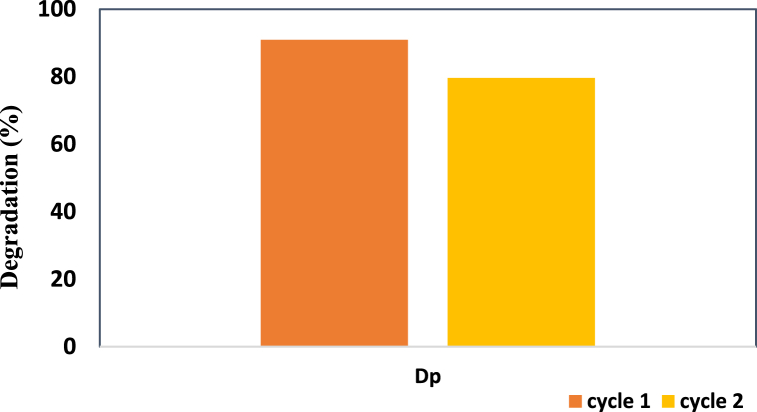
Reuse ability test for the ZnO-TiO_2_ nanocomposite.

The recycling efficiency of the ZnO-TiO_2_ nanocomposite was tested by running the sample in two cycles, and Fig. 10 demonstrates this phenomenon. The test was performed by using a 0.2 g sample with a 20 ppm (40 mL) dye concentration. With increasing cycle number, the degradation percentage started to decrease after one cycle, reaching 90.92 %, and became 79.60 % after the second cycle.

#### 3.3.10 Bandgap estimation

The energy required to excite an electron from the valence band to the conduction band is known as the band gap energy of a semiconductor. Precisely estimating the band gap energy is essential for evaluating the photophysical and photochemical characteristics of semiconductors. This parameter is most frequently used when discussing the photocatalytic properties of semiconductors. Using optical absorption spectra, Tauc established a technique in 1966 for determining the band gap energy of amorphous semiconductors [47].

**Fig. 11:**
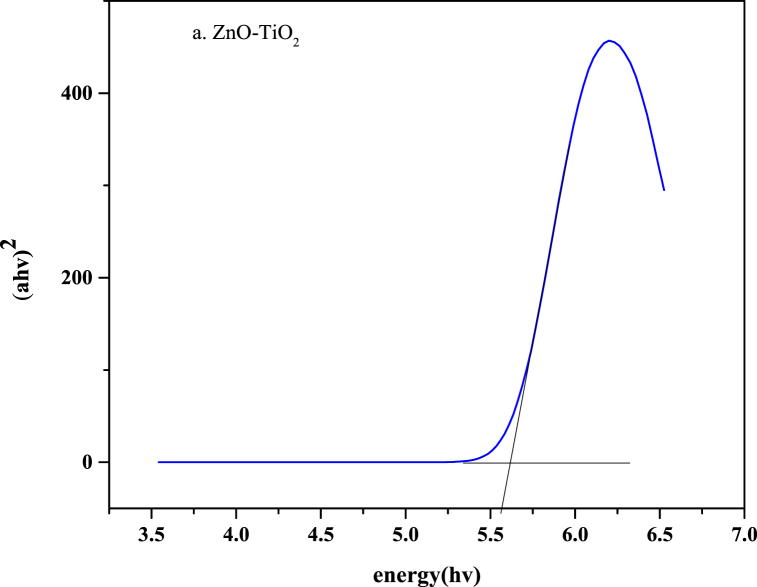
Bandgap energy for the a. ZnO-TiO_2_ nanocomposite.

The optical band gap of the ZnO-TiO_2_ nanocomposite was measured with the aid of Tauc's plot method while considering the data from a UV–visible spectrophotometer. While plotting the band gap graph in the original software, (αhv)^2^ and (hv) of Tauc's method equation were taken on the y-axis and x-axis, respectively (visualized in Fig. 11), in which α denotes the absorption coefficient, h is Planck's constant (h = 6.626 × 10^−34^ J s), and v is the frequency of photons [48]. The representation of Tauc's method equation (4) is as follows:

(4) αhϑ=(hϑ−Eg)

The optical band gap of the ZnO-TiO_2_ nanocomposite was 5.61.

### 3.4 SEM analysis

**Fig. 12:**
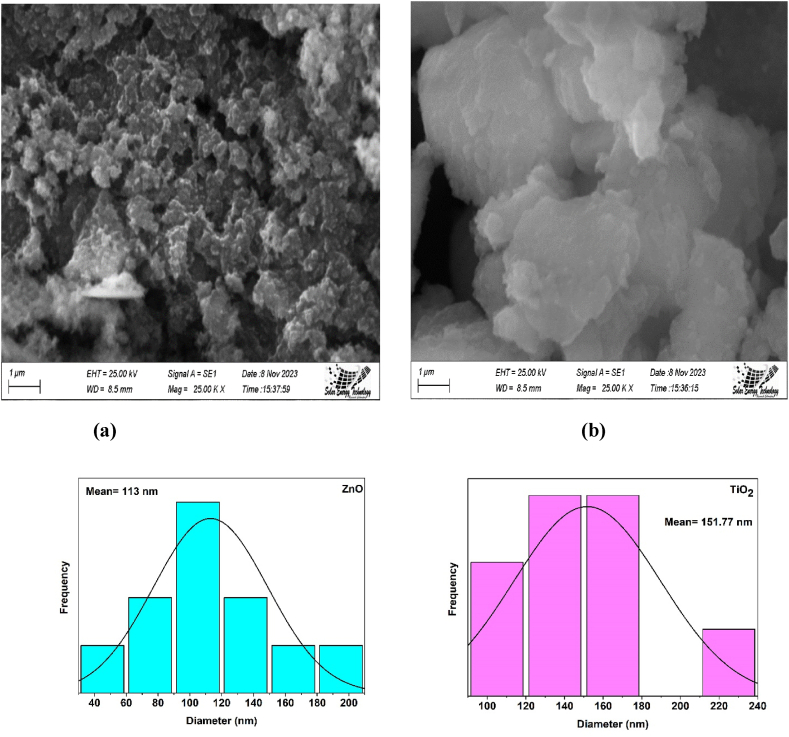
SEM analysis of (a) ZnO, (b) TiO_2_ and histogram analysis.

Using a scanning electron microscope (SEM), the surface morphologies of the synthesized ZnO and TiO_2_ nanoparticles were examined. The results are shown in Fig. 12.

Fig.12 (a) shows the randomly split, tightly packed, spherical shape on the surface of the ZnO nanoparticles, which were agglomerates of the nanocrystallites, and a similar result was previously observed [49]. The development of agglomerates may be a result of the high calcination temperature of ZnO. Using the Image J software and histogram analysis, the ZnO nanoparticle's particle size value was computed, and the result was 113 nm which complements some other literature [50]. The surface morphology of anatase TiO_2_ was explored through scanning electron microscopy. Fig. 12 (b) shows an SEM image of anatase TiO_2_, which reveals that anatase TiO_2_ forms aggregates with a sphere-like structure. Some undefined structures were also observed here. Also, the particle size of the TiO_2_ nanoparticle was obtained as 151.77 nm through image J software and histogram analysis. An earlier study showed a similar result [51,52].

### 3.5 Degradation mechanism of Congo red dye

Most studies conducted throughout the last two decades on the photocatalytic degradation of dyes have evaluated UV-active semiconductor photocatalysts. A summary of the indirect heterogeneous photocatalytic oxidation mechanism utilizing ZnO and TiO_2_ nanocomposite semiconducting materials is described below:

(a) Photoexcitation:

An irradiated semiconductor photocatalyst such as a ZnO-TiO_2_ composite will produce a photoelectron, which is promoted from the filled valence band to the empty conduction band, initiating a photocatalytic process. The photon energy (hv) absorbed in the absorber is either equal to or greater than the band gap of the semiconductor photocatalysts. A hole in the valence band (h_VB_^+^) is left behind by the excitation process. As a consequence, electron, and hole pairs (e^−^/h^+^) are produced as shown by equation (5).

(5) $$\text{ZnO}‐{\text{TiO}}_{2}+h\;\vartheta \;\to \;\text{ZnO}‐\;{\text{TiO}}_{2}\;\left(e^{‐}\left(\text{CB}\right)+h^{+}\left(\text{VB}\right)\right)$$

(b) Ionization of water:

After that, the water and photogenerated holes in the valence band combine to form the OH^•^ radical that is represented by equation (6).

(6) $$H^{+}\left(\text{VB}\right)+H_{2}O\;\to \;H^{+}+\text{OH}•$$

The OH• free radical, as an oxidizing agent, not only attacks organic pollutants but also microorganisms to remove contaminants [53].

(c) Oxygen ionosorption:

When surface-bound water or OH^−^ combines with photogenerated holes (h^+^VBs) to form hydroxyl radicals, equation (7) reveals that oxygen absorbs electrons during conduction (e_CB_^−^) to form anionic superoxide radicals (O_2_^•−^).

(7) $$O_{2}+e^{‐}\left(\text{CB}\right)\;\to \;O_{2^{{-}^{•}}}$$

(d) Protonation of superoxide:

The resultant superoxide (O_2_^•−^) is protonated, resulting in the formation of hydroperoxyl radicals (HO_2_^•^) and H_2_O_2_, which further break down into very reactive hydroxyl radicals (OH^•^). On the surface of photoexcited semiconductor photocatalysts, both oxidation and reduction reactions frequently occur. The reaction mechanism of photodegradation can be revealed by utilizing Equations 8–13.

(8) $$O_{2}^{‐.}+H^{+}\;\to \;H\text{OO}•$$

(9) $$2\;\text{HO}O•\to H_{2}O_{2}+O_{2}$$

(10) $$H_{2}O_{2}\;\to \;OH•$$

(11) $$\text{OH}•+\text{Congo}\;\text{red}\;\text{dye}\;\to \;{\text{CO}}_{2}+H_{2}O\;\left(\text{dye}\;\text{intermediates}\right)$$

(12) $$\text{Congo}\;\text{red}\;\text{dye}+h^{+}\left(\text{VB}\right)\;\to \;\text{oxidation}\;\text{products}$$

(13) $$\text{Congo}\;\text{red}\;\text{dye}+e^{‐}\left(\text{CB}\right)\;\to \;\text{reduction}\;\text{products}$$

### 3.6 Kinetics study

**Fig. 13:**
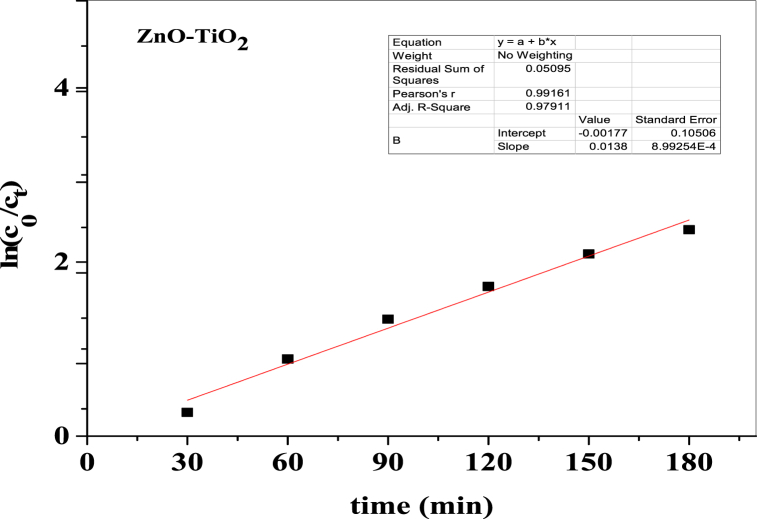
ln(c_0_/c_t_) vs time plot for photodegradation of Congo red dye using 0.2 g ZnO-TiO_2_ nanocomposite.

Numerous prior studies have demonstrated that the pseudo-first-order kinetics, which is based on the modified Langmuir–Hinshelwood model to account for reactions at a solid-liquid interface, most accurately describes the impact of the solution's initial concentration on the photocatalytic degradation rate of the majority of organic compounds [54,55]. The ZnO-TiO_2_ nanocomposite employed for the photocatalytic activity of Congo red dye exhibits pseudo-first-order kinetics. Equation (14) represents the rate of photocatalytic degradation of Congo red dye:

(14) $$\ln\left(\frac{c_{0}}{c_{t}}\right)=k.t$$

here, k denotes the pseudo-first-order reaction constant, t denotes time, C_0_ denotes the reactant's initial concentration and C_t_, the reactant's final concentration. An $\ln\left(\frac{c_{0}}{c_{t}}\right)$ vs time(t) plot is shown in Fig. 13 where $\ln\left(\frac{c_{0}}{c_{t}}\right)$ is along the y–axis and t along the x-axis. Based on this graph, the ZnO-TiO_2_ nanocomposite's first-order rate constant is 0.0138 min^−1^, and its strong correlation coefficient is close to 1 (0.97).

### 3.7 Langmuir adsorption isotherm

The Langmuir adsorption isotherm, which can be linear or non-linear, is frequently employed in this work to analyze the adsorption equilibrium data in the instance of Congo red dye adsorption onto the surface of the intended product. Equation (15) may be used to represent the Langmuir adsorption isotherm in its linear form [56],

(15) $$\frac{1}{q_{e}}=\frac{1}{K_{L}q_{\max}}\times \frac{1}{c_{e}}+\frac{1}{q_{\max}}$$

**Fig. 14:**
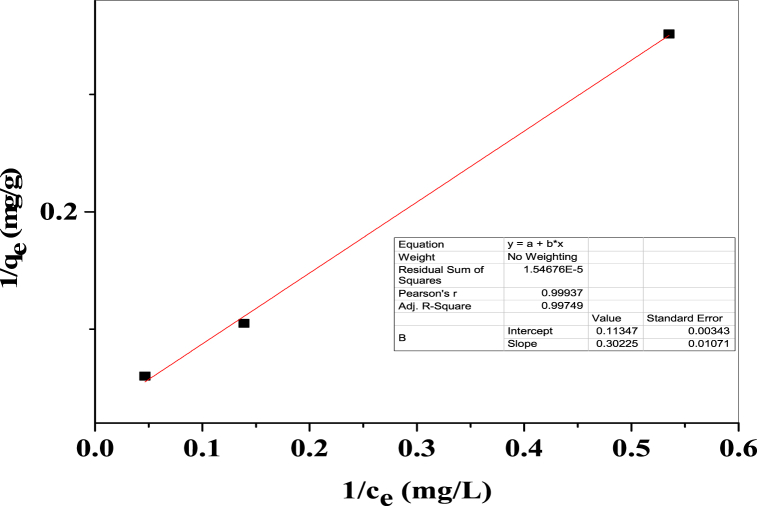
Graphical demonstration of linear Langmuir adsorption isotherm.

Here, c_e_ = Equilibrium concentration of dye in the solution in mg per L, q_e_ = Equilibrium concentration of dye adsorbed in mg per g, K_L_ = Langmuir equilibrium constant, q_max_ = Maximum adsorption capacity. Using Origin Pro 9 software, the slope and intercept value were calculated when 1/q_e_ along to y-axis and 1/c_e_ along to x-axis. Fig. 14 shows the graphical demonstration.

**Fig. 15:**
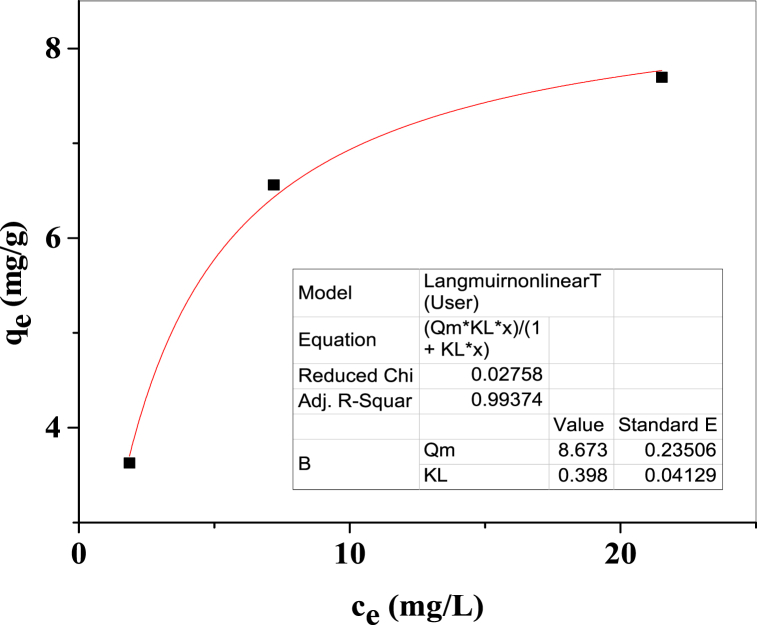
Graphical demonstration of non-linear Langmuir adsorption isotherm.

Equation (16) illustrates the non-linear form of the Langmuir adsorption isotherm equation [57],

(16) $$q_{e}=\frac{Q_{m}\;K_{L}c_{e}}{1+K_{L}c_{e}\;}$$

when, q_e_ and c_e_ were aligned with the y- and x-axes, respectively, the slope and intercept values were determined through Origin Pro 9 software which is displayed in Fig. 15. Depending on Langmuir linear and non-linear adsorption isotherm equation, the calculated values of isotherm parameters are enlisted in Table 2.

**Table 2:** Computed isotherm parameters.

Isotherm model	Curve Fitting	Parameters value	
		q_m_ (mg/g)	K_L_ (mg/L)
Langmuir	Linear	8.812	0.3754
	Non-linear	8.673	0.398

## 4 Conclusion

Due to the potential threat of textile wastewater to ecosystems, the safe disposal of industrial effluent is urgently needed, and ZnO-TiO_2_ nanocomposites have been proven to be excellent photocatalysts for the degradation of Congo red dye and other pollutants in wastewater. In this study, two metal oxide nanoparticles, viz., ZnO and TiO_2_ nanoparticles, were synthesized to degrade Congo red dye. While analyzing the photocatalytic performance, the ZnO-TiO_2_ nanocomposite showed superior photocatalytic activity. Several parameters, including the catalytic dose, initial dye concentration, duration of sample exposure to light, and pH, had substantial effects on the photocatalytic activity of the sample. A noteworthy result was achieved via a scavenger test that involved the use of IPA, EDTA, and DPPH to identify the reactive species responsible for degradation. After two cycles of reuse, the nanocomposite photocatalyst exhibited outstanding recycling efficiency and retained its catalytic properties. This study recorded a maximum 90.65 % degradation of Congo red dye when using an optimized 0.2 g photocatalyst with a 20 ppm (40 mL) dye concentration at pH 9 for 180 min of exposure. Therefore, our study recommends using this photocatalyst to treat wastewater in the textile sector.

## Data availability

The data will be made available upon request.

## CRediT authorship contribution statement

**Nusrat Jahan Tamanna:** Writing – original draft, Formal analysis, Data curation. **Md. Sahadat Hossain:** Writing – review & editing, Conceptualization. **Sumaya Tabassum:** Formal analysis. **Newaz Mohammed Bahadur:** Supervision. **Samina Ahmed:** Supervision.

## Declaration of competing interest

The authors declare that they have no known competing financial interests or personal relationships that could have appeared to influence the work reported in this paper.
