# Supplementary figures and images for: Easy and green synthesis of nano-ZnO and nano-TiO2 for efficient photocatalytic degradation of organic pollutants
Source: Heliyon. 2024 Sep 5;10(17):e37469. doi: 10.1016/j.heliyon.2024.e37469 (PMC11409113; doi:10.1016/j.heliyon.2024.e37469)

| 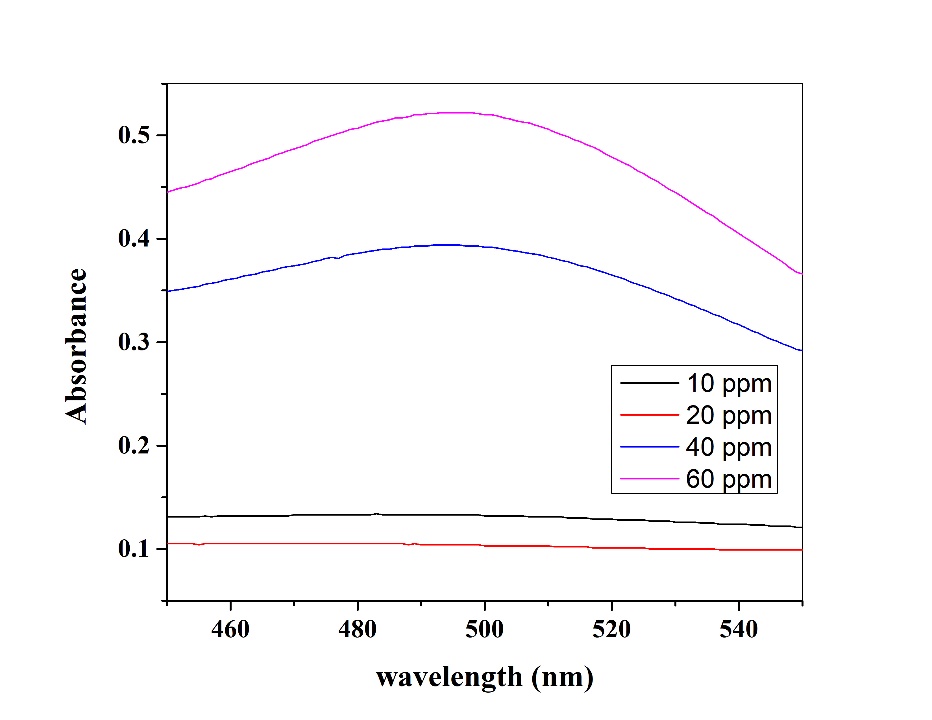 |
| --- |

**Fig. 16: Effect of several dye concentrations**

Supplement: Multimedia component 1 [file mmc1.docx]
